# Hydrocephalus as Possible Neurological Complication of COVID-19: A Case Report and Systematic Literature Review

**DOI:** 10.7759/cureus.41199

**Published:** 2023-06-30

**Authors:** Giulio Verrienti, Gianluigi Megliola, Antonio Colamaria, Tommaso Condò, Emilio Lozupone

**Affiliations:** 1 Department of Neurorehabilitation, Casa di Cura Villa Verde, Lecce, ITA; 2 Division of Neurosurgery, Riuniti Hospital, Foggia, ITA; 3 Department of Neuroradiology, Vito Fazzi Hospital, Lecce, ITA

**Keywords:** molecular mechanism, ace-2 receptor, aneurysmal subarachnoid haemorrhage, post-hemorrhagic hydrocephalus, covid 19

## Abstract

Coronavirus disease 2019 (COVID-19), caused by the severe acute respiratory syndrome coronavirus-2 (SARS-CoV-2), typically affects the respiratory system but can also present with neurological manifestations. Although some cases of hydrocephalus related to COVID-19 infection have been reported, a clear association between these two entities is not universally recognized yet.

Here, we report another interesting case of hydrocephalus in a 60-year-old man with a previous aneurysmal subarachnoid haemorrhage (aSAH) who tested positive for COVID-19. Secondly, we illustrate a systematic overview of the previously reported cases of hydrocephalus related to COVID-19 infection. Finally, in light of the literature, we discuss the supposed underlying mechanisms that could make the association between COVID-19 infection and hydrocephalus plausible.

## Introduction

Hydrocephalus is a condition in which an abnormal accumulation of cerebrospinal fluid (CSF) occurs within the cerebral ventricles. Several causes can promote hydrocephalus, including congenital malformations, trauma, subarachnoid haemorrhage (SAH) and tumor. However, recent epidemiologic studies have shown that the most common cause of hydrocephalus is infection (post-infectious hydrocephalus [pIH]) [1-2].

Posthemorrhagic hydrocephalus (pHC) is another common cause of hydrocephalus. pHC is a major complication after SAH, especially SAH due to aneurysm rupture, with an incidence ranging from 9% to 64% [3]. pHC is classified as acute (0 to three days post-SAH), subacute (four to 13 days post-SAH), or chronic (14 days post-SAH). Placement of a shunt system improves clinical outcomes in aSAH [4]. Several factors (e.g. high Hunt and Hess grade, presence of intraventricular haemorrhage, localisation of the ruptured aneurysm) predict the development of shunt-dependent hydrocephalus [5-6].

Recent research [1] has shown that pHC shares many similar issues with pIH (inflammatory and immune-mediated mechanisms, CSF hypersecretion by the choroid plexus, etc.).

Here, we report a rare case of hydrocephalus related to coronavirus disease 2019 (COVID-19) infection in a positively tested 60-year-old man with previous aSAH. A fascinating hypothesis for this case suggests that the COVID-19 infection acted as a precipitating factor for the development of the hydrocephalus in the context of a pre-existing vulnerability of the CSF system related to the previous SAH. 

## Case presentation

A 60-year-old man was brought to the emergency department (ED) due to unresponsiveness and respiratory distress. On admission, the patient was unconscious (Glasgow Coma Scale [GCS]: 4). In the emergency room, vital signs included temperature of 36.9 degrees Celsius, heart rate of 70 beats per minute, blood pressure of 180/90 mmHg. Past medical history was significant for chronic kidney failure, paroxysmal atrial fibrillation with previous ablation, essential hypertension and chronic obstructive pulmonary disease. After undergoing orotracheal intubation, the patient was moved to computed tomography (CT), which showed a diffuse SAH, tetra-ventricular hemorrhage and an intraparenchymal hematoma in the left frontal lobe (Fisher IV) due to rupture of an anterior communicating artery (AComA) aneurysm. The Hunt-Hess grade was V, while the assessed World Federation of Neurosurgical Societies (WFNS) score was 5. Consequently, an urgent endovascular treatment of the aneurysm and the subsequent external ventricular drainage (EVD) were performed. Endovascular treatment was preferred in relation to the poor clinical condition of the patient. The patient was admitted to the intensive care unit (ICU), where he was started on nimodipine 60 mg every four hours. The EVD was initially exchanged after six days due to insufficient drainage because of obstruction. The second EVD was removed on day 13, after 48 hours of EVD clamping and tomographic absence of enlarging ventricles in the brain CT control. At the suspension of the sedation the patient was only minimally responsive to painful stimuli. A percutaneous tracheostomy was carried out and a progressive weaning from mechanical ventilation was started. After six weeks, the patient was moved to our neurorehabilitation department.

At the baseline evaluation, the patient was alert, but unable to perform complex activities consistently or to communicate. However, he was able to protrude his tongue in a reproducible manner. An initial Revised Coma Recovery Scale score of 12 points was recorded. An intensive rehabilitation program was started in order to improve his consciousness and mobility. The patient underwent intensive multidisciplinary rehabilitation that included daily sessions of physiotherapy, speech and language therapy and neuropsychological rehabilitation. Various types of rehabilitation exercises were carried out according to the patient's health state and function. After intensive pulmonary rehabilitation, the tracheostomy was closed after decannulation. The patient started to communicate and eat normally. 

**Figure 1:**
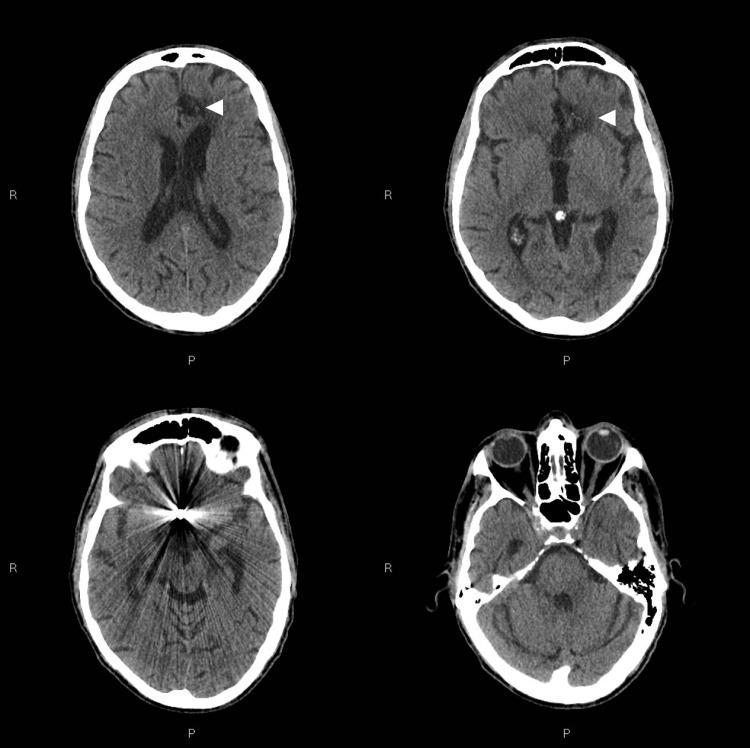
Six-month follow-up computed tomography (CT) scan after a diffuse subarachnoid haemorrhage due to rupture of an anterior communicating artery (AcomA) aneurysm The patient experienced a diffuse subarachnoid haemorrhage due to rupture of an anterior communicating artery (AcomA) aneurysm then treated by endovascular deposition of platinum coils. Six-month follow-up computed tomography scan demonstrated regular dimension of the ventricular system and a malacic transformation of an intraparenchymal hematoma in the medial aspect of the left frontal lobe (white arrowheads)

After a six-month high-intensity supervised training program, clinical conditions of our patient were consistently improved. The patient's functional assessment at this point of his clinical course demonstrated that he had attained voluntary control of all four limbs and he could perform a standing transfer with the assistance of one person. Furthermore, the patient could ambulate short distances with the assistance of two people; a head CT scan was performed before the presumptive home discharge (Figure 1).

**Figure 2:**
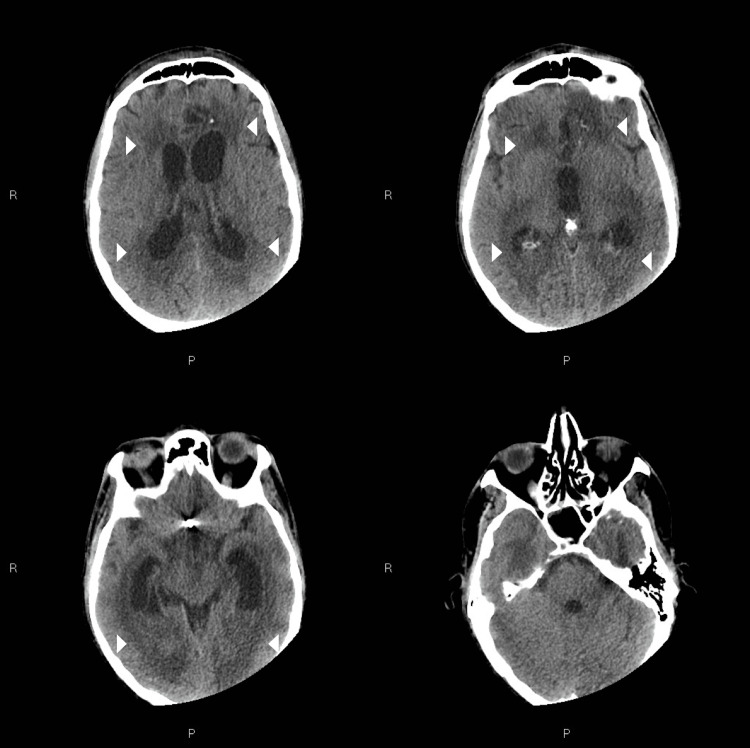
Non-enhanced computed tomography scan demonstrating the onset of acute hydrocephalus Four days after a positive COVID-19 test, neurological status of the patient rapidly worsened. Non-enhanced computed tomography scan demonstrated acute hydrocephalus, mainly involving supratentorial system. Periventricular hypodensity due to the transependymal cerebrospinal fluid resorption is demonstrated (white arrowheads).

Unfortunately, the patient’s condition worsened suddenly two weeks later, as he tested positive for COVID-19. The patient developed fever and respiratory symptoms after a few days. According to Italian Medicines Agency (AIFA) guidelines [7], treatment with remdesivir was attempted. Despite the adoption of various supportive treatments, including noninvasive ventilation (NIV) and antipyretic drugs, the patient showed a progressive consciousness disturbance. A brain CT showed the onset of acute hydrocephalus (Figure 2).

The patient was moved to the intensive care unit. Because of progressive CSF dynamic disorder an EVD was placed. While CSF cell count (5/5 cells) and glucose (0.5 g/L) were normal, proteins were notably enhanced (120 mg/dL). After three unsuccessful EVD clamping attempts, the EVD was exchanged with a definitive ventriculo-peritoneal (VP) shunt 16 days later. The clinical conditions of the patient remained critical. The appearance of adhesions in the ventricles, resulting in an abnormal CSF dynamic, led to the diagnosis of a compartmentalized, hypertensive hydrocephalus. Even if the patient was negative for COVID-19 at this time point, his neurological status was absolutely compromised. A second VP drain was placed, but the altered ventricular system anatomy due to formation of intracavitary adherences did not allow a physiologic CSF dynamic to be obtained. Because of poor neurological status and overall negative prognosis, a palliative setting was started. 

## Discussion

We report a case of a patient who had an aSAH caused by the rupture of an anterior communicating artery anerysm six months before severe COVID-19 infection. Despite his initial critical conditions after aSAH (GCS 4, Fisher IV, Hunt and Hess V, WFNS V), the patient experienced - over six months after aSAH - an excellent neurological recovery and he was planned to be discharged with outpatient continuation of neurological rehabilitation. Unfortunately, patient’s medical state worsened as he tested positive for COVID-19. Four days after testing positive for COVID-19, a non-enhanced CT scan demonstrated the new onset of acute hydrocephalus. The new diagnosed CSF dynamic disorder was not observed in a previous brain CT scan, which was performed two weeks earlier. Of note, the neurological state of the patient did not change between the first and the second CT examination; a progressive reduction in vigilance was otherwise observed as the patient developed fever and other COVID-19 symptoms. 

In the literature, it is well recognised that massive SAH may often lead to development of hydrocephalus [3,5-6], but, in our case report, a valuable hypothesis consists in the fact that COVID-19 infection could have played a role as a precipitating factor for the induction of hydrocephalus. In fact, even though we were not able to test CSF for COVID-19 antibodies due to laboratory limitations, the temporal correlation between the neurological impairment and the diagnosis of SARS-CoV-2 infection, as well as the radiologic evolution, make the hypothesis of an association between these two entities plausible. In particular, the previous SAH could have altered the CSF dynamic and the COVID-19 infection could have triggered the onset and the development of hydrocephalus.

**Figure 3:**
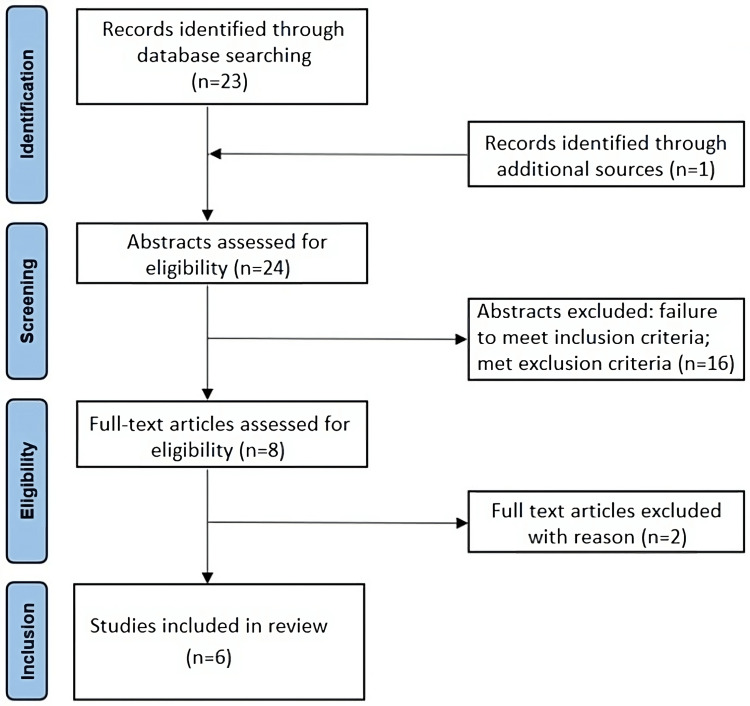
Preferred Reporting Items for Systematic Reviews and Meta-Analyses (PRISMA) flow chart According to PRISMA guidelines, a literature search (PubMed) was performed on 7 May 2023; we selected articles published from 1 January 2018 to 30 April 2023, using the following Medical Subject Headings (MeSH) terms: “Case report” AND “COVID 19” AND “hydrocephalus”. In addition, a backward search (checking the bibliography of identified papers) was conducted to identify any studies that were not retrieved using the main search strategy. The inclusion criteria were (1) articles published in English; (2) case reports regarding the relationship between hydrocephalus and COVID-19 infection; and (3) studies conducted in the above-reported period. In the main search strategy, the following exclusion criteria were adopted: (1) article types such as letters to the editor, reviews and meta-analyses; (2) case reports in pediatric age; (3) studies for which the complete text could not be found; and (4) articles not in English. Electronic and additional sources identified 24 references. A total of 16 of these were excluded after assessing their abstract, as they did not meet the inclusion criteria. Two studies [8,9] were removed after reading the full text, because, although they met inclusion criteria, the reported hydrocephalus was to refer to acute SAH. Thus, a total of six articles [10-15] were included.

However, hydrocephalus is not usually encountered among the neuro-COVID-19 complications and only a few cases reporting this association have been published. In order to identify the incidence of hydrocephalus during or after COVID-19 infection, we systematically searched PubMed for case reports and case series published in English in peer-reviewed journals, using the Medical Subject Heading (MeSH) terms “Case report” AND “COVID 19” AND “hydrocephalus”. The main features of our search procedure and the Preferred Reporting Items for Systematic Reviews and Meta-Analyses (PRISMA) flow chart are reported in Figure 3. Literature review yielded six studies (four case reports and two case series), which are summarized in Table 1. 

**Table 1:** Studies included in the systematic review Literature review yielded six studies, four case reports [10-13] and two case series [14,15] iNPH: idiopatic normal pressure hydrocephalus; ADHD: attention-deficit/hyperactivity disorder

First author [reference]	Year	Case report/series description
Saini [10]	2022	A patient with a history of recent COVID-19 infection who presented with chronic progressive headaches with nausea, vomiting, and blurry vision over 2 weeks
Vasconcelos TMF [11]	2022	A case of iNPH 2 months after acute COVID-19 infection, in a patient without other risk factors
Oredipe O [12]	2022	A 25-year-old woman presenting with severe hydrocephalus and acute stroke
Badar F [13]	2023	A 48-year-old male with a past medical history of ADHD, hypertension, and hyperlipidemia who developed typical symptomatology of iNPH with cognitive impairment, gait dysfunction, and urinary incontinence after a mild COVID-19 infection
Torelli G [14]	2023	2 patients with a known iNPH condition, in which neurological symptoms suddenly worsened, requiring hospitalization, without any evident precipitating cause
Dai X [15]	2023	Three cases of hydrocephalus that appear or worsen after COVID-19 infection. The common features of all three cases were: (i) all three patients were young women; (ii) all three cases were confirmed with neo-coronavirus infection at the same time; (iii) all three cases presented with mild respiratory symptoms after neo-coronavirus infection; (iv) all three cases presented with clinical manifestations and worsening of hydrocephalus within 2-4 weeks after neo-coronavirus infection; and (v) all three cases underwent ventriculi-abdominal shunt after a positive fluid release test, with significant relief of symptoms after surgery

According to our review, hydrocephalus onset during or after COVID-19 infection shows in adults an equal distribution among genders (five female, four male) and ages (age interval: 20-79). In almost half of the identified cases, the diagnosis of hydrocephalus was made concomitantly with the diagnosis of COVID-19 infection. A delayed hydrocephalus development after COVID-19 infection has been also reported. In almost all cases of our review a VP shunt placement was performed (Table 2). 

**Table 2:** Patient characteristics and clinical outcomes iNPH: idiopatic normal pressure hydrocephalus; ADHD: attention-deficit/hyperactivity disorder; EVD: external ventricular drainage, VP: ventriculo-peritoneal

Ref.	Sex, Age	Co-morbidities	Time from COVID-19 infection and hydrocephalus onset (1)	Symptoms	Interventions	Outcome and follow up
[10]	M/36	Hypertension	2 weeks	Nausea, vomiting, blurry vision, intermittent horizontal diplopia	EVD placement and suboccipital craniotomy, a subarachnoid web was microsurgically resected	Home discharge
[11]	M/45	Bipolar disorder; type II diabetes mellitus	8 months	Imbalance and progressive gait disorder;memory impairment, executive dysfunction	High-volume (40 ml) lumbar tap test followed by VP Shunt placement	Home discharge. After 30 days significant improvement of initial symptoms was reported
[12]	F/25	-	Acute	Acute headache and vomiting, followed by consciousness impairment	EVD placement	Discharge to a rehabilitation center
[13]	M/48	ADHD; hyper-tension; hyper-lipidemia	5 months	Headaches, vertigo, gait instability, lethargy, confusion, blurry vision changes	EVD placement followed by VP Shunt placement	Discharge to a rehabilitation facility
[14]	M/79	History of iNPH	Acute	Worsening of the neurological status with walking inability for severe gait instability	VP Shunt placement was refused by the patient	Not reported
[14]	F/20	Chronic migraine	Acute	Progressive consciousness impairment	VP Shunt placement	Home discharge with complete resolution of symptoms
[15]	F/35	-	4 weeks	Severe headache	VP Shunt placement	Home-discharge with complete resolution of symptoms
[15]	F/29	Previous diagnosis of aqueduct stenosis with supratentorial hydrocephalus	Acute	Headache, nausea, vomiting, confusion, urinary incontinence	VP Shunt placement	Home discharge with complete resolution of symptoms
[15]	F/36	-	4 weeks	Gait instability, headache, nausea, vomiting	VP Shunt placement	Home discharge with complete resolution of symptoms
(1): or worsening of a previous diagnosed hydrocephalus.						

The belief that COVID-19 infection could act as a triggering factor for the development of CSF dynamic disorder (eventually leading to deterioration of previously diagnosed hydrocephalus), is supported by some case reports included in our review [14-15]. Torelli et al. [14] described a case of a 79-year-old unvaccinated man with a history of idiopathic normal pressure hydrocephalus who experienced a worsening of his neurological status during acute COVID-19 infection. Dai et al. [15] reported a case of a 29-year-old female with a history of non-VP shunt-requiring congenital stenosis of the middle cerebral aqueduct. In this case report, the acute COVID-19 infection led to manifestation of Hakim's trias. A VP shunt was placed and the symptoms were significantly relieved after the operation.

The exact underlying mechanism of hydrocephalus induced by COVID-19 infection is not completely understood and many theories were discussed in the included studies of our review. A proposed explanation is related to the SARS-CoV-2 virus's ability to bind to human cells via the angiotensin-converting enzyme 2 (ACE2) receptor. The ACE2 receptor is widely expressed in different tissues (e.g. respiratory epithelia, kidneys, liver, blood vessels), but also in the central nervous system (CNS). In particular, ACE2 receptors are abundantly expressed in the choroid plexus of the lateral ventricles [16]. The proposed interaction between the virus and the choroid plexus could potentially lead to an alteration of the microscopical structure of the choroid plexus with subsequent establishment of arachnoid webs [10], resulting in an alteration of the CSF flow dynamic. Other possible mechanisms underlying the pathogenesis of hydrocephalus caused by COVID-19 include the ‘Trojan horse’ mechanism of immune cells and the systemic reaction leading to a hypercoagulable state. According to the Trojan horse mechanism, the immune-mediated cytokine storm, triggered by SARS-CoV-2 infection of multiple immune cells, may also lead to inflammation of the arachnoid villi, resulting in reduced absorption of CSF and subsequent hydrocephalus [16,17]. Furthermore, the systemic reaction occurring during COVID-19 infection could lead to a hypercoagulable state of the blood system. In some patients, the presence of hypercoagulability may lead to venous congestion and abnormal CSF flow dynamic, resulting in hydrocephalus [11].

Including case reports and case series to describe the relationship between COVID-19 infection and the development of hydrocephalus, an internal limitation of our systematic review is the inherent weaknesses of case reports as a source of evidence. In fact, case reports and case series do not provide strong causal evidence in comparison with other designs, such as analytical observational studies. In particular, one major limitation of case studies consists in the fact that it is often difficult to generalize findings from the reported case to the wider population. On the other hand, the major advantages of case reports and case series are the ability to highlight new observations, therefore generating hypotheses. In particular, in the absence of other evidence, case reports and case series may provide valuable information to researchers and clinicians [18].

The main purpose of a systematic review of case reports and case series - as in our research - is to accumulate scientific data about rare conditions, providing, at the same time, stronger evidence in confirming or denying the supposed hypothesis. With regard to our research, this review represents the first attempt to systematically identify and examine cases of hydrocephalus related to COVID-19 infection. As a result, we found only a few case reports and a limited number of patients reported in the literature. In this context, we believe there is too little evidence to establish a clear and definitive association between these two entities. Also, it should be observed that the incidence of hydrocephalus related to COVID-19 infection may be underestimated due to the simultaneous presence of systemic symptoms (e.g. high fever), which may mask neurological symptoms. For this reason, we believe that future research should adopt more rigorous methodologies, such as large-scale observational studies, to establish a clearer association between COVID-19 and hydrocephalus. Finally, we believe that healthcare professionals should be aware of this possible association in order to manage, in due time, likely reversible conditions such as the development of CSF dynamic disorder. 

## Conclusions

In conclusion, we report a suspected case in which COVID-19 infection could have altered the CSF dynamic, leading to hydrocephalus. In addition, we systematically searched for other published cases regarding the association between COVID-19 and hydrocephalus. To the best of our knowledge, this is the first systematic review on this topic. Finally, we discussed the underlying pathogenic mechanisms for this association.

Based on the available literature, an association between hydrocephalus and COVID-19 could be neither established nor excluded. In fact, the evidence on this topic is limited to a few anecdotal reports and a generalisation may be not possible. The real incidence of CSF dynamic disorders during or after COVID-19 infection may be underestimated. For this reason, future research with a more rigorous methodology (i.e. large-scale observational studies) should be undertaken to provide conclusive evidence.
